# Quantification of Lower Limb Kinematics During Swimming in Individuals with Spinal Cord Injury

**DOI:** 10.3390/s25133950

**Published:** 2025-06-25

**Authors:** Melina Giagiozis, Sabrina Imhof, Sibylle Achermann, Catherine R. Jutzeler, László Demkó, Björn Zörner

**Affiliations:** 1 Spinal Cord Injury Center, University Hospital Balgrist, 8008 Zürich, Switzerland; sabrina.imhof@balgrist.ch (S.I.); sibylle.achermann@paraplegie.ch (S.A.); laszlo.demko@balgrist.ch (L.D.); bjoern.zoerner@paraplegie.ch (B.Z.); 2 Department of Health Sciences and Technology (D-HEST), ETH Zurich, 8092 Zürich, Switzerland; catherine.jutzeler@hest.ethz.ch; 3 Swiss Institute of Bioinformatics (SIB), 1015 Lausanne, Switzerland; 4 Swiss Paraplegic Research, 6207 Nottwil, Switzerland; 5 Swiss Paraplegic Centre, 6207 Nottwil, Switzerland

**Keywords:** spinal cord injury, swimming kinematics, breaststroke, lower limbs, inertial sensors, IMU

## Abstract

Spinal cord injuries (SCI) often result in impaired motor functions. To quantify these impairments, swimming patterns were analyzed in individuals with SCI. Water provides a unique rehabilitation environment where buoyancy supports weight-bearing activities and can reveal deficits that might otherwise go unnoticed. Data were collected of 30 individuals with chronic, motor-incomplete SCI and 20 healthy controls during breaststroke swimming on a kickboard. Using eight wearable inertial sensors attached to the lower limbs, we captured detailed kinematic data. Spatiotemporal parameters were then calculated and compared between the two groups to assess differences in swimming patterns. An analysis of the parameters revealed significant differences in velocity (*p* < 0.001, ε^2^ = 0.476) and distance per stroke (*p* < 0.001, ε^2^ = 0.516), indicating decreased swimming speeds in individuals with SCI compared to controls. Furthermore, individuals with SCI demonstrated a reduced range of motion (RoM) in the ankle (*p* = 0.003, ε^2^ = 0.516) and knee joints (*p* = 0.041, ε^2^ = 0.142). The limited RoM likely contributes to the shorter distance covered per stroke. These observations underscore the impact of SCI on functional capabilities. The developed algorithm holds promise for enhancing the assessment of motor deficits after neurological injuries.

## 1. Introduction

A spinal cord injury (SCI) can significantly impact an individual’s ability to control and coordinate their movements [1]. The extent of these deficits varies depending on the location and severity of the lesion in the spinal cord and usually includes impaired motor and sensory functions [2]. Approximately half of all SCI are classified as functionally incomplete with some motor or sensory function preserved below the level of injury, potentially allowing for the recovery of walking function [3]. However, gait patterns of individuals with SCI may differ from those of healthy individuals, exhibiting impairments in rhythm, speed, or a combination of gait characteristics, often accompanied by compensatory movements [4].

The current gold standard for movement analysis is marker-based motion capture, providing both spatiotemporal and kinematic information [5,6]. However, most optical motion capture systems require a complex and costly setup of cameras and equipment, often restricting their use to controlled environments like gait laboratories. Promising alternatives are inertial measurement units (IMUs), wearable movement sensors [7]. These sensors have proven to be a reliable tool in assessing human motion and complementing clinical assessments [8,9]. IMUs are cost-effective, easy to operate, and suitable for use in water, making them ideal for both clinical settings and sports [10]. They were previously employed in studies for functional gait assessments such as the 2 min walking test (2MWT) and the 6 min walking test (6MWT) [4,11].

In cases where gait function is significantly impaired, assessing movements in water alongside traditional gait tests may provide additional insights into the locomotor abilities of an individual with SCI. Aquatic therapy is often recommended for the rehabilitation of motor functions [12]. One advantage of water-based therapy is buoyancy, which offloads body weight and reduces pressure on the joints [13]. The water provides greater safety compared to land-based exercises by minimizing the risk of falling, allowing individuals with SCI to move in ways they might otherwise not be able to. Additionally, the drag force of water offers resistance, which can strengthen muscles and improve coordination [14]. Currently, there is limited research examining the kinematics of leg movements during swimming, and existing studies primarily focus on the swimming patterns of healthy individuals with varying skill levels (experts vs. recreational swimmers) [15,16,17]. Swimming kinematics are typically evaluated using video analysis [18]. In recent years, interest in using IMUs to monitor swimming performance has grown, as they have been shown to capture the temporal phases of breaststroke movement [19] and are considered a reliable tool for performance assessment in aquatic environments [18]. However, their precision and efficacy during physical activities in water, particularly in individuals with neurological injury, requires further analysis.

The aim of this study was to perform a comprehensive analysis of swimming patterns of individuals with chronic, motor-incomplete SCI and healthy controls. All participants swam in an indoor pool whilst wearing eight sensors attached to their lower limbs and back. Furthermore, demographical data and clinical scores were collected to evaluate the relationship between the sensor-derived parameters and established clinical characteristics. We hypothesized that sensor-derived swimming parameters can be used to identify functional motor deficits and, thereby, enhance conventional clinical assessments.

## 2. Materials and Methods

### 2.1. Cohort Definition: Inclusion and Exclusion Criteria

Data were collected from individuals with chronic (>6 months), motor-incomplete SCI and healthy controls. All included individuals exhibited impaired walking functions but could walk a minimum of ten meters without assistance or assistive devices and were able to swim breaststroke. Furthermore, individuals with SCI were required to be between 18 and 80 years of age with a neurological level of injury (NLI) above T12. Similarly, all healthy participants were between 18 and 80 years old. Participants were excluded if they had a history of major cardiac or pulmonary conditions, a current major depression or psychosis, or any orthopedic or neurological conditions that could affect their breaststroke movement.

### 2.2. Study Design and Data Collection

**Figure 1:**
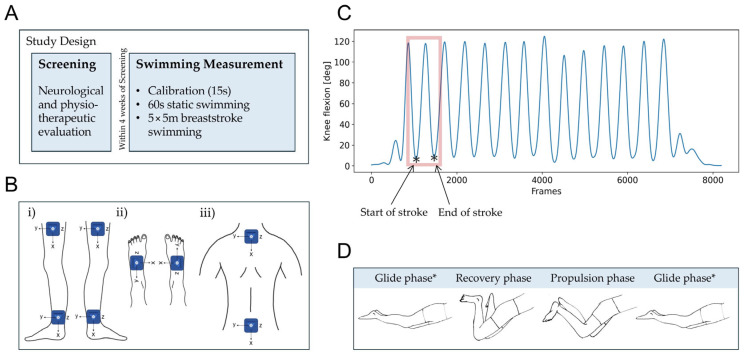
(**A**) Study design including a screening phase with a thorough neurological and physiotherapeutic evaluation, followed by a swimming measurement. (**B**) Inertial sensor setup with the local coordinates of each module. IMUs were placed on (**i**) the lateral left and right shanks and ankles, (**ii**) the top of the left and right feet, and (**iii**) the lower cervical and lumbar spine. (**C**) Knee flexion/extension angles during breaststroke swimming. When the knee reaches maximum extension (indicated by *), the previous stroke cycle ends and the next begins. (**D**) Leg movement during a cycle of breaststroke swimming. Abbreviations: deg: degrees.

This study consisted of an initial screening visit for participants with SCI, as shown in Figure 1A. This visit involved a comprehensive neurological and physiotherapeutic evaluation conducted by qualified clinicians. During this evaluation, clinical scores were assessed, including the American Spinal Injury Association Impairment Scale (AIS) [20], the NLI, and the mobility subscale of the Spinal Cord Independence Measure (SCIM III) [21]. Additionally, a distinction between the more and less impaired legs was made for each individual based on the neurological examination [22]. For this, motor scores were compared between the left and right legs; if these were identical, ataxia measures were considered, followed by sensory scores. Demographic data, including age, sex, and BMI, were also collected for all participants.

Within four weeks of the screening, individuals with SCI completed a swimming assessment (Figure 1A). Healthy controls participated only in this assessment. Participants were familiar with breaststroke swimming but received no training as part of this study. The protocol included a short calibration phase, where participants stood motionless for 15 s. During this calibration, the sagittal orientation of all wearable sensors was measured, which was later used to correct for any offset inclination angles caused by body curvature. To acclimate to the water environment and the exercise setup, participants swam in place on a stationary kickboard (104 × 55 cm) supporting their upper body. A therapist held the kickboard while participants were instructed to swim breaststroke for 60 s, maintaining the smoothest stroke possible. Finally, individuals with SCI were instructed to swim a total of five 5 m lengths of breaststroke on a kickboard as fast as possible (maximum speed) to establish comparable kinematic conditions across participants. This distance was chosen to ensure feasibility for participants with more severe impairments while allowing the capture of sufficient stroke cycles to analyze representative swimming patterns across all participants. The purpose of the kickboard was to stabilize individuals with SCI in the water and prevent compensatory arm movements, allowing for a focus solely on lower limb kinematics. As a result, participants relied exclusively on their legs for propulsion. If unable to complete five lengths, individuals with SCI were encouraged to swim as many lengths as they were able. Healthy controls swam five lengths of 5 m following the same protocol, in addition to swimming at their individual preferred speed (comfort speed) for five lengths of the same distance.

All participants swam in an indoor pool (length, width, depth: 16.7 × 8.0 × 1.31 m) wearing eight inertial sensors (ZurichMOVE, Zurich, Switzerland) attached with flexible straps to their lower limbs and back, as depicted in Figure 1B. The specific placement of the sensors was on the right and left thighs, one-third above the *lateral epicondyle* (on the line between *lateral epicondyle* and *trochanter*) on the right and left ankles, directly above the *lateral malleolus*; on the right and left feet, dorsally in the middle of the line between the *medial malleolus* and the *fifth metatarsal*; on the upper back, below the *processus spinosus* of the *C7 vertebra*; and on the lower back, above the *sacrum* at the level of the *iliac crest*. The IMU modules (MPU-9250, 35 × 35 × 12 mm, 18 g), each equipped with a tri-axial accelerometer and a tri-axial gyroscope, were wirelessly time-synchronized and recorded at a sampling frequency of 200 Hz. The sensor attached to a participant’s lower back was selected as master for synchronization, while all other sensors functioned as slaves.

### 2.3. Sensor-Derived Swimming Parameters

A novel algorithm was developed in Python (version 3.7, MacOS) to extract metrics from raw IMU data, based on the orientation of the modules. The accuracy of the module orientations was validated using a marker-based optical motion capture system (Vicon Motion Systems Ltd., Oxford, UK) operating at 200 Hz, with 32 cameras, and processed in Nexus 2.10.0 (Vicon, Oxford, UK). Two healthy controls each performed three trials of 30 s of dry-land swimming on parallel bars in a gait laboratory. The lower limb kinematics were recorded with both the Vicon system and the IMUs attached, as shown in Figure 1B. The two systems were synchronized with the use of a piezo element [8]. When a Vicon measurement was initiated, the piezo was triggered and its movements recorded by a reference IMU. Three markers were placed at the corners of each sensor, indicating the direction of the x-axis and y-axis. From these, the z-axis was determined based on the right-hand rule to form a complete coordinate system. Data were then collected from the sensors while their positions were tracked using optical motion capture. Changes in the orientation of individual sensor modules, calculated from the IMU data, were compared to those obtained from the optical motion capture system. The average range of motion over a stroke cycle as well as the root mean squared error (RMSE) and mean signed difference (MSD) were calculated for the ankle, knee, and hip joint angles to assess discrepancies between the two measurement systems. While the RMSE measured the overall magnitude of the difference, the MSD revealed any systematic bias by indicating whether IMUs consistently over- or underestimated the joint angles.

Swimming cycles were segmented based on knee angles in the sagittal plane [23]. The start of a stroke cycle was defined as coinciding with the maximum knee extension of the relevant leg, which corresponded to a minimum in the knee angle in the sagittal plane, as shown in Figure 1C. A knee extension of zero degrees was measured when the leg was fully extended. The legs remained straight during the glide phase, after which they were drawn toward the body as the knees and hips flexed, transitioning into the stroke’s recovery phase. The swimming cycle, or stroke, ended as the legs extended once again, returning to the glide phase, ready to initiate the next stroke cycle, as illustrated in Figure 1D. All spatiotemporal swimming parameters, as summarized in Table 1, were calculated as the mean over all swimming strokes. For select parameters, the standard deviation was also computed across all strokes as a measure of their variability.

Joint angles were derived using the Madgwick filter, a popular algorithm for sensor fusion in motion tracking applications [24]. This filter combines data collected by the IMUs to estimate orientation with high accuracy and reduced drift. The orientation of each sensor frame *S* relative to the Earth frame *E* at any given time point *t* can be denoted in the form of quaternions $q_{ES}\left(t\right).$ The orientation of each sensor was further adjusted to ensure that the x-axis pointed downwards and the y-axis pointed forwards for all sensors in the Earth frame. Following the right-hand rule, the z-axis was aligned laterally, pointing to the right of the sensor in the Earth frame. The right thigh sensor served as the reference for aligning all other sensors within a common reference frame.

We could then derive the corresponding rotation matrix for each quaternion $q_{ES}(t),$ denoted $R_{ES}\left(t\right)=R(q_{ES}\left(t)\right).$ A sensor-to-segment calibration was performed to account for any offset inclination angles caused by the curvature of the body segments that the sensors were attached to. Finally, we calculated the rotation matrix between sensors $S_{1}$ and $S_{2}$, or their corresponding body segments, as follows:

(1) $$R_{S_{1}S_{2}}\left(t\right)={R_{ES_{1}}\left(t\right)\;}^{T}\times R_{ES_{2}}\left(t\right),$$

where $R_{ES_{1}}\left(t\right)$ and $R_{ES_{2}}\left(t\right)$ are the rotation matrices representing the orientation of sensors $S_{1}$ and $S_{2}$ relative to the Earth frame *E* at time point *t*. Here, *T* denotes the transpose and × indicates matrix multiplication.

Finally, angles of the ankle, knee, and hip joints were obtained by calculating the Euler angles from the relevant rotation matrices. The angle of a joint corresponded to the Euler angle derived from the rotation matrices describing the rotations between the body segments connected to that joint. Hip flexion/extension and hip abduction/adduction involve the thigh rotating in relation to the lower back around the z-axis and y-axis, respectively. Knee flexion/extension is determined by the movement of the shank relative to the thigh around the z-axis, while ankle dorsiflexion/plantarflexion is defined by the movement of the foot in relation to the shank around the z-axis.

The joint angle data were then segmented into individual stroke cycles and interpolated to ensure uniformity, with each cycle comprising 100 joint angle data points. Angle–angle plots, or cyclograms, were derived from the sagittal joint angles (ankle–knee and knee–hip). Once centered on the origin, the shape of an individual’s cyclogram can be compared to the healthy reference using the sum of squared distances (SSD), a measure of deviation from the healthy range [25]. The smaller the SSD, the more closely the cyclogram aligns with the physiological shape. To assess the inter-leg asymmetry, the SSD between the left and right legs was calculated. This asymmetry SSD quantified the difference between the average left and right cyclograms. The consistency of these cyclograms across all stroke cycles was measured using the angular component of the coefficient of correspondence (ACC), a value that ranges from 0 to 1 [26]. The closer the ACC is to 1, the higher is the consistency in joint angles throughout all stroke cycles. The range of motion (RoM) of the ankle, hip, and knee joints was also obtained. This range represents the extent of movement achieved in each joint in degrees and is a critical measure in assessing joint function and mobility [27]. Moreover, a phase shift, also known as phase dispersion, was calculated to assess the synchronicity between the legs during each stroke [22]. A phase shift of 0% signified perfect synchronization, with both legs entering the glide phase, or start of the stroke cycle, simultaneously, while a higher value suggested a discrepancy or shift in the timing of the legs’ movements.

To allow for the comparison of parameters with different units, they were normalized using z-scores. *Z*-score normalization transforms each parameter x into a standard score Z by subtracting the mean μ and dividing by the standard deviation σ of the healthy control data:

(2) $$Z=\frac{x-\mu }{\sigma }.$$

This process converts the parameters to a common scale, allowing for direct comparison across different units [28]. By normalizing to the healthy data, we ensured that deviations from the healthy range were standardized. This means that a z-score of 0 indicated a value equal to the healthy mean, while positive or negative z-scores indicated values above or below the healthy mean, respectively, with the magnitude of the z-score reflecting the number of standard deviations from the mean.

### 2.4. Statistical Analyses

Swimming parameters (Table 1) were analyzed for healthy controls at both comfortable and maximum speeds to establish a performance baseline. Subsequently, the latter was compared to the swimming patterns at maximum speed of individuals with chronic, motor-incomplete SCI, aiming to identify potential deficits. Statistical comparisons were conducted using the Kruskal–Wallis test (α = 0.05) with a Holm–Bonferroni correction for multiple testing. Age and body mass index (BMI) were controlled for in the analysis to account for their potential influence on performance outcomes.

Furthermore, a cluster analysis was performed on the swimming parameters of individuals with SCI to identify characteristic swimming patterns. For this purpose, a principal component analysis was applied to the scaled parameters to reduce the dimensionality of the dataset. The optimal number of principal components was determined based on their cumulative explained variance [29]. Then, a k-means clustering was performed on the principal components [30]. Using the Elbow Method, the number of clusters that minimized the within-cluster sum of squares was selected. To validate this choice, we additionally applied the Silhouette Score and Gap Statistic, both well-established methods suitable for datasets of this size [31]. The resulting clusters were compared in terms of demographic data and clinical scores. Due to the non-normality of the data, a Mann–Whitney U test was selected for the continuous variables (e.g., SCIM) and a Fisher’s Exact Test was selected test for the categorical variables (e.g., NLI). Furthermore, the swimming parameters that best differentiated the clusters were identified. Specifically, the parameters that contributed the most to each principal component and differed significantly between clusters were selected using the Kruskal–Wallis test (α = 0.05) with a Holm–Bonferroni correction for multiple testing. Pairwise post hoc comparisons were conducted using the Mann–Whitney U test (α = 0.05) to compare the clusters. The statistical analysis was performed in Python (version 3.7, MacOS).

## 3. Results

### 3.1. Subjects

Data were collected from 30 individuals with chronic (>6 months), motor-incomplete SCI and 20 healthy controls. The demographic and clinical characteristics of all participants are summarized in Table 2. Individuals with SCI were significantly older (Mann–Whitney U test: U = 110.0, *p* < 0.001, r = −0.532), with a mean age of 55.4 ± 14.0 years, whereas the healthy controls had a mean age of 37.3 ± 12.7 years. Additionally, females comprised 30% of the SCI cohort and 50% of the control group. The average BMI among individuals with SCI was 25.1 ± 4.0 kg/m^2^, which was significantly higher compared to the 22.1 ± 2.2 kg/m^2^ among healthy participants (Mann–Whitney U test: U = 154.0, *p* = 0.004, r = −0.408). All participants with SCI were classified with an AIS [20] grade of D. The NLI varied among individuals with SCI, including 12 individuals with cervical SCI and 18 with thoracic injuries. The etiology was primarily non-traumatic (e.g., ischemic), with 19 instances, while the remaining 11 were traumatic injuries. The SCI cohort had an average score of 35.2 ± 5.2 in the mobility subscale of the SCIM III [21]. All participants were familiar with breaststroke swimming, and none swam beyond a recreational level. On average, the time since they last swam using this style was 1.28 ± 2.55 years.

### 3.2. Algorithm

**Figure A1:**
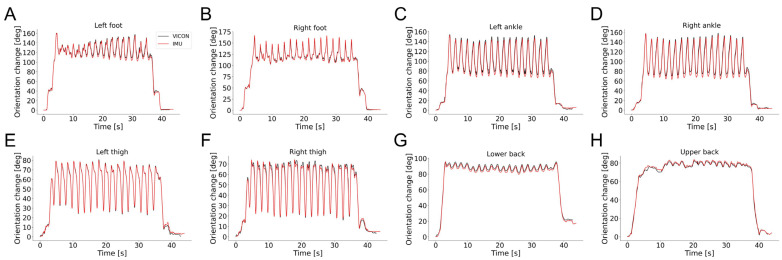
Comparison of the orientation changes estimated from the IMU and optical motion capture (VICON) data during a 40 s dry-land breaststroke swimming trial of a healthy participant. Orientation change was calculated as the rotation angle required to align the estimated orientation at each time point with that at the initial time point. Subfigures show orientation changes at individual sensor locations: (**A**) Left foot, (**B**) right foot, (**C**) left ankle, (**D**) right ankle, (**E**) left thigh, (**F**) right thigh, (**G**) lower back, (**H**) upper back. Abbreviations: deg: degrees, s: seconds.

**Figure A2:**
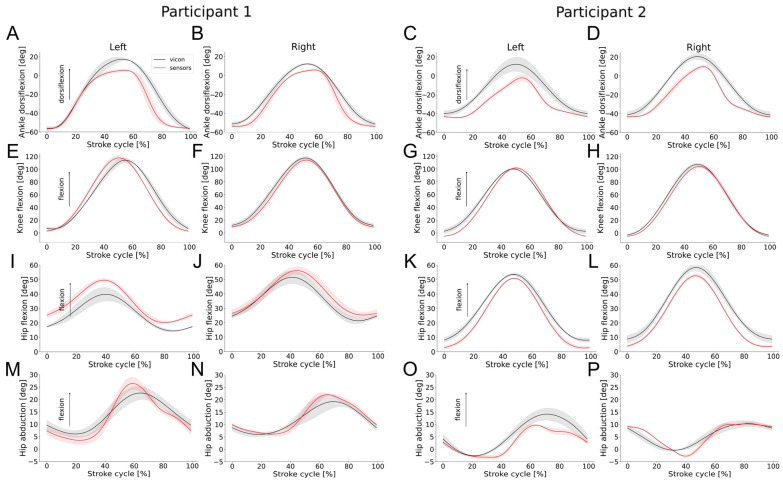
Average and standard deviation of ankle, knee, and hip angular excursions during a stroke cycle of dry-land breaststroke swimming of two healthy controls, using two motion capture systems (IMUs and Vicon). The start of a stroke cycle was defined as coinciding with the maximum knee extension of the relevant leg. Subfigures show joint angles for each participant and side as follows: (**A**–**D**) Ankle dorsiflexion, (**E**–**H**) knee flexion, (**I**–**L**) hip flexion, (**M**–**P**) hip abduction. Abbreviations: deg: degrees.

Orientation estimates and calculated joint angles of two participants were compared between two motion capture systems (IMU and Vicon). While the orientation changes estimated from the IMU data followed those calculated from the Vicon data (Figure A1 in Appendix A), the Vicon system generally recorded higher RoM values compared to the IMUs, as indicated by mostly negative MSDs (Figure A2, Table A1 in Appendix A). The RMSE was largest for the ankle joint, while the knee and hip joint angles showed less pronounced differences, with an RMSE of mostly less than 5 degrees. The ankle’s susceptibility to larger errors can be attributed to challenges in sensor placement and the curvature of the foot. Nonetheless, the observed differences between the two systems remained below the clinically significant minimum detectable change of 5 to 10 degrees [32] for all joints, affirming the reliability of IMUs to measure breaststroke swimming kinematics.

### 3.3. Swimming Patterns of Healthy Participants

**Figure 2:**
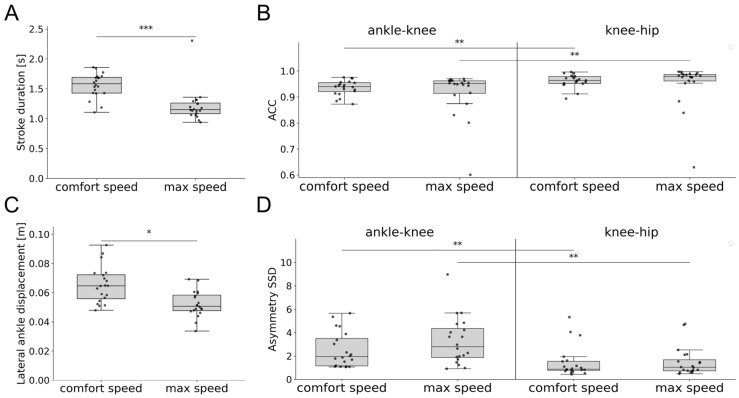
Swimming parameters of healthy controls swimming at comfort speed compared to maximum speed, including: (**A**) stroke duration, (**B**) ACC of the knee–ankle and hip–knee angles, (**C**) lateral displacement of the ankle, and (**D**) asymmetry SDD of the knee–ankle and hip–knee angles. Asterisks represent significant differences with * *p* < 0.05, ** *p* < 0.01, and *** *p* < 0.001, according to the Mann–Whitney U test with a Holm–Bonferroni correction. Abbreviations: ACC: angular component of coefficient of correspondence; SSD: sum of squared distances.

On average, healthy controls swam 0.11 m/s faster when instructed to swim at maximum speed compared to comfort speed. They exhibited no differences in RoM, ACC, and asymmetry SSD at different speeds. However, stroke duration (Kruskal–Wallis test: H(1) = 17.8, *p* < 0.001, ε^2^ = 0.431) and lateral ankle displacement (Kruskal–Wallis test: H(1) = 11.4, *p* = 0.022, ε^2^ = 0.268) differed significantly (Figure 2). Furthermore, significant differences in ACC and asymmetry SSD were observed between proximal (knee–hip) and distal joints (ankle–knee). The ACC of proximal joints was significantly higher compared to distal joints at both comfort speed (Kruskal–Wallis test: H(1) = 10.0, *p* = 0.003, ε^2^ = 0.257) and maximum speed (Kruskal–Wallis test: H(1) = 11.8, *p* = 0.001, ε^2^ = 0.303). Similarly, the asymmetry SSD of proximal joints was significantly lower than that of distal joints at comfort speed (Kruskal–Wallis test: H(1) = 10.4, *p* = 0.003, ε^2^ = 0.266) and maximum speed (Kruskal–Wallis test: H(1) = 11.8, *p* = 0.001, ε^2^ = 0.303).

### 3.4. Swimming Patterns of Individuals with SCI

**Figure 3:**
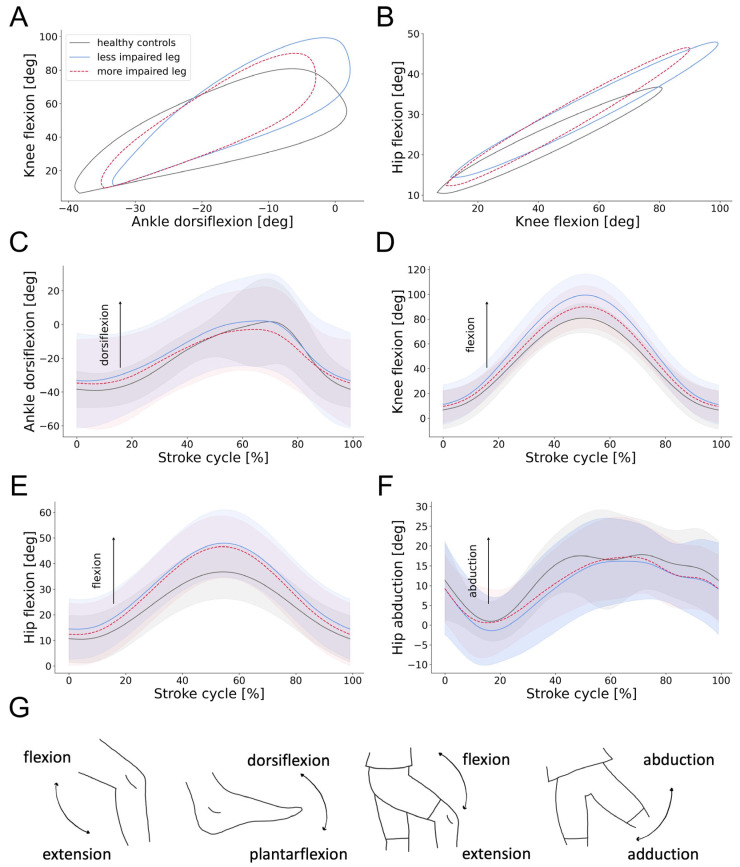
Joint angles of individuals with SCI compared to healthy controls. (**A**,**B**) Angle–angle plots (cyclograms) depicting intra-limb coordination of the ankle, knee, and hip joints during breaststroke swimming. (**C**–**F**) Average and standard deviation of ankle, knee, and hip angular excursions during a stroke cycle of individuals with SCI and healthy controls. The start of a stroke cycle is defined as coinciding with the maximum knee extension of the relevant leg. (**G**) Illustration of all relevant joint movements. Abbreviations: deg: degrees.

**Figure 4:**
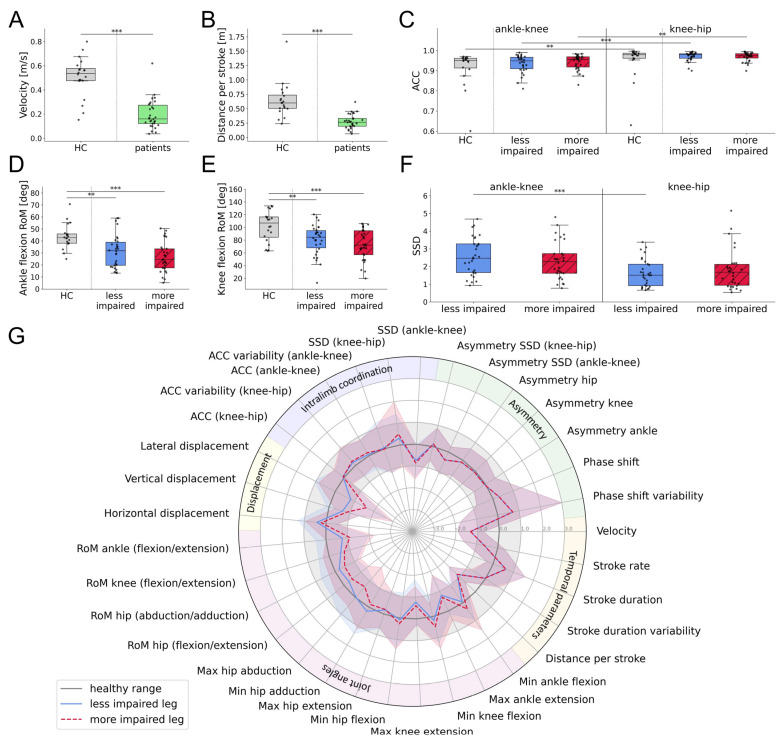
Swimming parameters of individuals with SCI compared to healthy controls, both swimming at maximum speed, including: (**A**) velocity, (**B**) distance per stroke, (**C**) ACC of the sagittal ankle–knee and knee–hip angles, (**D**,**E**) RoM of the ankle and knee joints, and (**F**) SSD of the sagittal ankle–knee and knee–hip angles. (**G**) Average swimming profile of individuals with SCI compared to healthy controls, both swimming at maximum speed. Swimming parameters were normalized with respect to the healthy range. Asterisks represent significant differences with ** *p* < 0.01, and *** *p* < 0.001, according to the Mann–Whitney U test with a Holm–Bonferroni correction. Abbreviations: SCI: spinal cord injury, HC: healthy controls, RoM: range of motion, ACC: angular component of coefficient of correspondence; SSD: sum of squared distances; max: maximum; min: minimum.

For the healthy cohort, differences between the more and less dominant legs were investigated, but no statistically significant disparities were found (Table A2 in Appendix A). Therefore, the average was calculated between both legs and compared to individuals with SCI. The cyclogram shapes were comparable between individuals with SCI and healthy controls (Figure 3A,B). The angular excursions of the ankle, knee, and hip joints throughout a stroke cycle are presented in Figure 3C–F. Individuals with SCI exhibited a greater flexion in the hip and knee joints at 40% to 60% of the stroke cycle. This observation was particularly pronounced in the knee flexion of the less impaired leg. In contrast, ankle plantarflexion/dorsiflexion and hip abduction/adduction remained within the healthy range throughout the stroke cycle. As shown in Figure 4, significant differences between individuals with SCI and healthy controls were observed in velocity (Kruskal–Wallis test: H(1) = 24.3, *p* < 0.001, ε^2^ = 0.476), distance per stroke (Kruskal–Wallis test: H(1) = 26.3, *p* < 0.001, ε^2^ = 0.516), and RoM of the ankle (Kruskal–Wallis test: H(2) = 18.3, *p* = 0.003, ε^2^ = 0.516) and knee joints (Kruskal–Wallis test: H(2) = 13.1, *p* = 0.041, ε^2^ = 0.142). While the more impaired leg exhibited greater deviations from the healthy RoM, differences were also present in the less impaired leg. The ACC was comparable across the two cohorts, though the previously observed decrease in ACC when moving from proximal to distal body segments was more pronounced in individuals with SCI. In particular, the ACC of proximal and distal joints differed significantly for both the more impaired (Kruskal–Wallis test: H(1) = 41.17, *p* = 0.002, ε^2^ = 0.449) and less impaired leg (Kruskal–Wallis test: H(1) = 41.17, *p* < 0.001, ε^2^ = 0.521). Similarly, the SSD was significantly higher in distal compared to proximal segments for the less impaired leg (Kruskal–Wallis test: H(1) = 17.0, *p* < 0.001, ε^2^ = 0.286). The overall swimming profiles of individuals with SCI and healthy controls, with parameters normalized to the healthy data, are summarized in Figure 4G.

### 3.5. Characterization of Swimming Clusters

**Figure 5:**
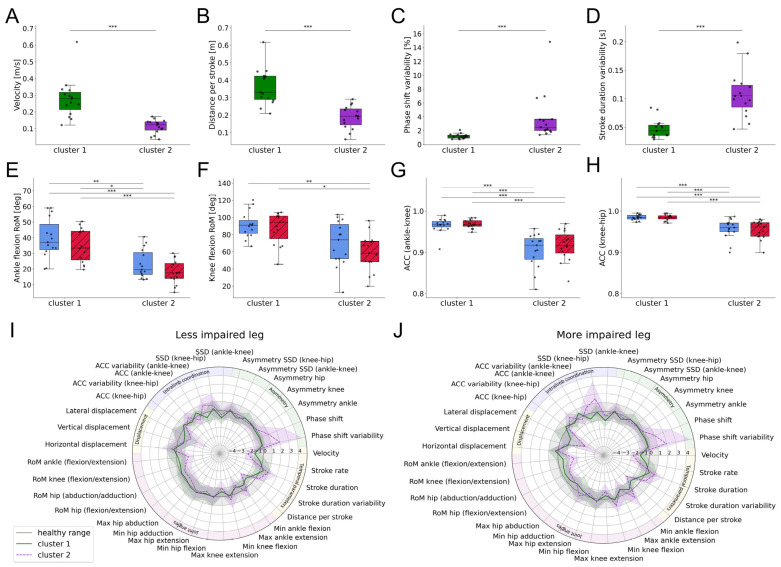
Swimming parameters of the SCI clusters including: (**A**) swimming velocity, (**B**) distance per stroke, (**C**) variability in percentage phase shift, (**D**) stroke duration variability, (**E**,**F**) RoM of the ankle and knee joints, and (**G**,**H**) ACC of the ankle–knee and knee–hip angles. (**I**,**J**) Average swimming profile of clusters for the more and less impaired legs compared to healthy controls, both swimming at maximum speed. Swimming parameters were normalized with respect to the healthy range. Asterisks represent significant differences with * *p* < 0.05, ** *p* < 0.01, and *** *p* < 0.001, according to the Mann–Whitney U test with a Holm–Bonferroni correction. Abbreviations: RoM: range of motion; ACC: angular component of coefficient of correspondence; SSD: sum of squared distances; max: maximum; min: minimum.

All individuals with SCI were clustered according to their swimming parameters to identify common patterns. The first six principal components explained 74% of the variance in the dataset and were used for the clustering algorithm. Moreover, the within-cluster sum of squares was minimized with two distinct clusters (Table A3 in Appendix A). This choice was further supported using both the Silhouette Score and Gap Statistic. The five most important features of each principal component were selected and tested for significant differences between the two clusters. This process identified eight discerning swimming parameters, which are presented in Figure 5A–H.

Cluster 1 showed higher swimming velocities (Kruskal–Wallis test: H(1) = 17.7, *p* < 0.001, ε^2^ = 0.597) and distance per stroke (Kruskal–Wallis test: H(1) = 17.4, *p* < 0.001, ε^2^ = 0.585) compared to cluster 2. Additionally, cluster 1 had a lower variability in percentage phase shift (Kruskal–Wallis test: H(1) = 18.8, *p* < 0.001, ε^2^ = 0.635) and stroke duration (Kruskal–Wallis test: H(1) = 16.7, *p* < 0.001, ε^2^ = 0.560). The RoM in the ankle (Kruskal–Wallis test: H(3) = 26.9, *p* < 0.001, ε^2^ = 0.426) and knee joints (Kruskal–Wallis test: H(3) = 16.8, *p* < 0.001, ε^2^ = 0.247) was significantly higher in cluster 1, as was the ACC of the ankle–knee (Kruskal–Wallis test: H(3) = 35.8, *p* < 0.001, ε^2^ = 0.586) and the knee–hip angles (Kruskal–Wallis test: H(3) = 34.2, *p* < 0.001, ε^2^ = 0.556). The swimming profiles of both clusters, with parameters normalized to the healthy cohort, are depicted in Figure 5I,J.

Demographically, the clusters did not differ significantly. Additionally, the average time since participants last performed breaststroke swimming was comparable between the clusters, with participants in cluster 1 reporting an average of 1.25 ± 2.54 years and those in cluster 2 reporting 1.30 ± 2.55 years. However, cluster 1 showed a significantly higher SCIM III Mobility compared to cluster 2 (Mann–Whitney U test: U = 166.0, *p* = 0.026, r = 0.405). Further analysis revealed that some members of cluster 1 required crutches or a cane to walk distances of both 10–100 m and >100 m as well as to manage stairs. Individuals in cluster 2 faced similar mobility challenges, but to a greater extent. The need for crutches or a cane was more common in cluster 2, and several members required this aid even for indoor mobility. Furthermore, some members of cluster 2 used an electric or manual wheelchair for distances of both 10–100 m and >100 m.

## 4. Discussion

In this work, we presented a data-driven quantification of the lower limb kinematics of 30 individuals with chronic, motor-incomplete SCI and 20 healthy subjects during breaststroke swimming. By applying signal processing and machine learning techniques to sensor-derived swimming data, we aimed to (i) compare the swimming patterns of healthy participants at comfortable and maximum speeds, (ii) evaluate the differences between the swimming patterns of individuals with SCI and healthy controls, and (iii) classify individuals with SCI into distinct clusters based on their swimming characteristics. Healthy participants exhibited consistent swimming patterns across different speeds, though differences in stroke duration and lateral ankle displacement were observed. Individuals with SCI displayed significant deviations in velocity, distance per stroke, and RoM compared to healthy controls. Additionally, a clustering analysis identified two distinct groups within the SCI cohort, differentiated by swimming performance metrics such as velocity, phase shift, and RoM, providing insights into the diverse functional impacts of SCI on swimming ability.

### 4.1. Swimming Patterns of Healthy Controls and Individuals with SCI

We selected breaststroke swimming due to its clinical and technical advantages. The complex movement pattern and numerous degrees of freedom involved in breaststroke make it particularly suitable for detecting motor deficits in the lower limbs. Moreover, its distinct and consistent motion allows for a detailed biomechanical analysis using inertial sensors [33]. The relatively slow speed and well-defined phases of breaststroke swimming enable more precise movement tracking, reducing variability compared to faster strokes such as freestyle [18]. Furthermore, as one of the most common swimming styles, participants are generally familiar with breaststroke.

Swimming parameters of healthy participants were compared at two different speeds. No significant changes were observed in RoM, asymmetry, or coordination based on the sensor-derived swimming parameters. However, there were significant differences in stroke duration and lateral displacement of the ankles. These observations suggest that healthy individuals can increase their swimming speed through quicker strokes, without compromising their inter- or intra-limb coordination. Quicker strokes can enhance propulsion and forward momentum, while reducing lateral ankle displacement suggests improved control and energy efficiency. The maintained RoM, symmetry, and coordination of healthy subjects at fast swimming speeds could be attributed to their neuromuscular control and core stability, which allow for precise and efficient movements [34]. Understanding these mechanisms can benchmark swimming parameters that describe functional impairments for individuals with SCI.

Swimming parameters of participants with SCI were compared to those of healthy controls, both swimming at their maximum speed. Individuals with SCI demonstrated lower swimming velocities, shorter distances per stroke, and a reduced RoM in the ankle and knee joints compared to healthy controls. They appeared to swim more slowly than controls primarily because they covered less distance per stroke, likely due to impaired RoM in the ankle and knee joints. Moreover, individuals with SCI showed a more pronounced decline in coordination from proximal to distal body segments. SCI often leads to decreased muscle strength and coordination [35], which may affect the ability to generate effective propulsion in the water. Furthermore, SCI can limit joint mobility, further decreasing stroke efficiency [27]. These impairments may contribute to the differences in swimming speeds and RoM observed compared to healthy participants. The amplified challenges in distal joints suggest that impairments in proximal segments, such as the hip and trunk, have a cascading effect on the distal segments. These results emphasize the need for targeted rehabilitation strategies that focus not only on the most affected distal joints but also on the proximal. Enhancing proximal stability and strength could potentially improve distal joint control and coordination in individuals with SCI.

By identifying specific motor deficits in the lower limbs, sensor-derived swimming profiles can enhance conventional clinical assessments of individuals with SCI. They offer objective insights into strength, joint mobility, and coordination that are uniquely captured during breaststroke swimming.

### 4.2. Characterization of Swimming Clusters

A clustering algorithm based on the sensor-derived swimming parameters separated the participants with SCI into two distinct clusters. Cluster 1, despite exhibiting lower swimming speeds compared to healthy participants, demonstrated the ability to maintain synchronization between the more and less impaired legs. In fact, individuals in this cluster can achieve a level of inter-limb coordination comparable to that of healthy individuals. Additionally, cluster 1 exhibited a high level of pattern consistency, indicating a robust intra-limb coordination that mirrors healthy movement patterns. In terms of rehabilitation, the focus for cluster 1 should be on maintaining and enhancing these abilities and further improving speed through targeted strength and endurance training.

In contrast, participants in cluster 2 face more significant challenges, with lower speeds, reduced inter-limb coordination, and larger deviations from healthy patterns. Additionally, this cluster exhibited reduced intra-limb coordination, reflecting less stable movement patterns. This implies that participants in cluster 2 encountered more pronounced challenges in maintaining symmetric and consistent swimming patterns compared to those in cluster 1, indicating a higher degree of impairment. Rehabilitation programs for cluster 2 should prioritize improving motor control and coordination through, for example, specific exercises targeting the neuromuscular system [36]. Additionally, personalized approaches considering the specific impairments and challenges of each individual are crucial for effective rehabilitation [37].

The swimming profiles of both clusters are visualized in Figure 5. The distinct differences between them highlight the heterogeneity within the SCI population [38]. These differences are further underscored by the mobility subscale of SCIM III, with cluster 1 showing significantly higher scores compared to cluster 2. The mobility subscale of the SCIM III provides valuable insights into the ability of individuals with SCI to perform various mobility-related activities, though it does not directly assess swimming capabilities. Individuals in cluster 2 rely more heavily on mobility aids than members of cluster 1, indicating a higher level of impairment. This difference indicates that the degree of mobility impairment influences swimming capabilities, with individuals experiencing greater mobility impairments exhibiting more pronounced deviations from healthy swimming patterns. Specifically, difficulties with indoor mobility and the need for a wheelchair correlate with diminished swimming abilities. Therefore, improving these abilities through targeted rehabilitation programs should also enhance swimming patterns in individuals with SCI. Conversely, one might argue that training swimming abilities to improve coordination, strength, and joint mobility can positively impact overall mobility and SCIM III scores.

### 4.3. Limitations

The sample size in this study is reasonable given the rarity of SCI but can still be considered small in the broader context of data science and statistical analysis, where larger samples are typically preferred to ensure more robust and reliable findings [39]. Smaller sample sizes can introduce greater variability and limit the ability to detect subtle differences or relationships within the data. However, the primary aim of this study was to demonstrate the value of our method, for which the sample size was sufficient. Nonetheless, larger, multi-center studies should be conducted to confirm our results.

Furthermore, we found differences in demographic characteristics between individuals with SCI and healthy controls, particularly in age and BMI. Although these variables were statistically controlled for, residual effects cannot be entirely ruled out. Nevertheless, the observed group differences in kinematic parameters remained robust after statistical adjustment, suggesting that these findings are unlikely to be solely driven by demographic factors.

The developed IMU-based algorithm relies on sufficient knee flexion and extension for accurate stroke detection, which can be challenging for individuals with SCI [40]. In this study, two subjects were unable to achieve the necessary knee flexion in one or both knees, resulting in their exclusion from the analysis. Due to this reliance on adequate knee movement, the algorithm’s accuracy in detecting strokes may have been affected, particularly for individuals with more severe impairments. While this may introduce a potential sampling bias, the aim of the present study was to characterize swimming kinematics during breaststroke swimming. Therefore, individuals who were unable to perform recognizable breaststroke movements had to be excluded from the analysis.

### 4.4. Clinical Applications and Future Work

This work is a step toward the use of IMUs for the assessment of the rehabilitation progress of individuals with SCI, demonstrating the potential of wearable sensors under different conditions and in diverse environments, such as overground walking, wheeling, and swimming. IMUs offer a non-invasive, cost-effective solution for the continuous monitoring of movement, with the potential to deliver more precise and personalized assessments than traditional methods. By enabling detailed, objective feedback, these technologies can help advance and structure aquatic therapy while also supporting remote performance monitoring outside clinical settings. This is particularly valuable for individuals with limited access to specialized therapy, for instance, due to geographic or financial constraints. Having established the feasibility of IMU-based swimming analysis in individuals with SCI, future research should focus on longitudinal applications of the developed tool to monitor changes in motor function associated with rehabilitation and therapeutic interventions. By tracking and analyzing changes in lower limb kinematics over time, one can gain a more profound understanding of the mechanisms of motor recovery and can more accurately evaluate the effectiveness of therapeutic interventions designed to enhance mobility and functional independence.

## 5. Conclusions

We quantified the swimming patterns of individuals with chronic, motor-incomplete SCI as well as healthy controls, providing a comprehensive overview of their lower limb kinematics during this motor task. The developed algorithm was able to accommodate the wide range of motor deficits present in individuals with SCI, demonstrating its potential as a tool for monitoring motor functions throughout the entire rehabilitation process. The precise measurement of specific kinematic parameters, such as joint angles and coordination, enables the identification of an individual’s unique motor deficits. This allows clinicians to tailor therapeutic interventions more effectively by targeting the specific impairments that require the most attention. Consequently, the algorithm holds promise for improving our understanding of specific impairments and enhancing the customization of physical rehabilitation programs to meet the unique needs of individuals.

## Figures and Tables

**Table 1:** Swimming parameters.

Swimming Parameter (Features)	Unit	Description
Velocity (mean)	Meters per second (m/s)	Swimming speed
Stroke rate (mean)	Strokes per minute (strokes/min)	Stroke cadence
Stroke duration (mean, SD)	Seconds (s)	Time between consecutive glide phases
Distance per stroke (mean)	Meters (m)	Distance covered with each stroke
Joint angles (min, max)	Degrees (deg)	Angles of the ankle, knee, and hip joints
Range of motion (mean)	Degrees (deg)	Range of motion of the ankle, knee, and hip joints
Ankle displacement (mean)	Meters (m)	Lateral, horizontal, and vertical displacements of the ankle during the propulsion phase
ACC (mean, SD)	Range [0, 1]	Intra-limb coordination
SSD (mean)	Degrees squared (deg^2^)	Deviation from the healthy pattern of intra-limb coordination
Asymmetry (mean)	Percentage (%)	Percentage difference in joint angles between left and right legs
Asymmetry SSD (mean)	Degrees squared (deg^2^)	Deviation of intra-limb coordination between left and right legs
Phase shift (mean, SD)	Percentage (%)	Inter-limb coordination

Abbreviations: SD: standard deviation; min: minimum; max: maximum; ACC: angular component of coefficient of correspondence; SSD: sum of squared distances.

**Table 2:** Characteristics of individuals with SCI and healthy controls with corresponding *p*-values calculated using the Mann–Whitney U test for continuous and the Fisher’s Exact Test for categorical variables.

Cohort	Individuals with SCI	Healthy Controls	*p*-Values
Number, *n*	30	20	
Age [years], mean (SD)	55.4 (14.0)	37.3 (12.7)	<0.001
Sex [female], *n* (%)	9 (30.0)	10 (50.0)	0.235
BMI [kg/m^2^], mean (SD)	25.1 (4.0)	22.1 (2.2)	0.004
AIS D, *n* (%)	30 (100.0)		
NLI, *n* (%)	12 (40.0) cervical 18 (60.0) thoracic		
Etiology, *n* (%)	11 (36.7) traumatic 19 (63.3) non-traumatic		
SCIM Mob., mean (SD)	35.2 (5.2)		

Abbreviations: BMI: body mass index; AIS: ASIA Impairment Scale; NLI: neurological level of injury; SCIM Mob.: mobility subscale of the Spinal Cord Independence Measure III.

## Data Availability

The anonymized data used in this study are available upon reasonable request, in compliance with the General Data Protection Regulation (EU GDPR). The code used to process the data and calculate the results can be accessed on our GitLab repository at https://gitlab.ethz.ch/BMDSlab/publications/sci/sci-swimming-analysis (accessed on 16 May 2025). Sample data of one healthy participant are available at https://zenodo.org/records/15344749 (accessed on 16 May 2025).

## Abbreviations

The following abbreviations are used in this manuscript:

ACC	Angular component of coefficient of correspondence
AIS	American Spinal Injury Association Impairment Scale
BMI	Body mass index
IMU	Inertial measurement unit
MSD	Mean signed difference
NLI	Neurological level of injury
RMSE	Root mean squared error
SCI	Spinal cord injury
SCIM	Spinal Cord Independence Measure
SSD	Sum of squared differences
2MWT	2 min walking test
6MWT	6 min walking test

## Appendix A

**Table A1:** Means and standard deviations of the RoM measured with Vicon and IMUs of the ankle (plantarflexion/dorsiflexion), knee (flexion/extension), and hip (flexion/extension and abduction/adduction). The RMSE and MSD were calculated between Vicon and IMUs.

Joint	RoM IMUs	RoM Vicon	RMSE	MSD	RoM IMUs	RoM Vicon	RMSE	MSD
Participant 1	Left				Right			
Ankle (plantarflexion/dorsiflexion)	62.9 (3.5)	69.5 (7.8)	7.9 (0.8)	−1.5 (0.1)	60.6 (0.7)	64.0 (2.0)	5.0 (0.4)	−1.7 (0.3)
Knee (flexion/extension)	119.4 (1.0)	125.0 (22.6)	7.3 (0.3)	1.1 (0.9)	108.7 (2.0)	105.4 (2.5)	4.0 (1.7)	−1.2 (0.3)
Hip (flexion/extension)	30.8 (2.4)	26.2 (3.5)	2.5 (0.3)	−0.5 (0.3)	31.2 (3.4)	30.4 (3.4)	4.4 (0.3)	−3.4 (0.1)
Hip (abduction/adduction)	18.0 (2.0)	17.5 (1.7)	5.9 (0.9)	4.2 (0.7)	14.0 (2.8)	13.5 (3.6)	2.3 (0.2)	0.6 (0.5)
Participant 2	Left				Right			
Ankle (plantarflexion/dorsiflexion)	53.2 (7.1)	52.6 (9.2)	9.6 (1.1)	−8.6 (1.2)	57.4 (3.0)	62.9 (4.9)	9.4 (0.1)	−7.7 (0.1)
Knee (flexion/extension)	103.7 (3.8)	96.7 (3.0)	4.4 (0.4)	−3.3 (0.3)	111.6 (2.2)	109.4 (3.0)	4.3 (0.3)	−2.8 (0.3)
Hip (flexion/extension)	50.1 (1.7)	46.6 (2.1)	3.5 (0.3)	−3.1 (0.4)	51.16 (1.1)	50.61 (4.6)	4.1 (0.4)	−3.0 (0.3)
Hip (abduction/adduction)	15.1 (0.2)	16.9 (2.1)	2.8 (0.6)	2.0 (0.7)	14.1 (1.7)	11.3 (0.8)	2.0 (0.1)	0.1 (0.5)

Abbreviations: RoM: range of motion; deg: degrees; RMSE: root mean squared error; MSD: mean signed difference.

**Table A2:** Swimming parameters of healthy controls for the dominant and non-dominant legs, compared using the Mann–Whitney U test with a Holm–Bonferroni correction.

Swimming Parameter	Features	Dominant Leg	Non-Dominant Leg	*p*-Values
Stroke duration [s], mean (SD)		1.22 (0.3)	1.22 (0.3)	1.000
Stroke duration variability [s], mean (SD)		0.08 (0.07)	0.1 (0.09)	1.000
Joint angles [deg], mean (SD)	Min ankle dorsi-/plantarflexion	−9.5 (14.6)	−7.8 (14.4)	1.000
	Max ankle dorsi-/plantarflexion	34.5 (13.6)	35.6 (14.6)	1.000
	Min knee flexion/extension	−6.0 (13.2)	−2.1 (20.5)	1.000
	Max knee flexion/extension	100.6 (20.3)	94.8 (25.5)	1.000
	Min hip flexion/extension	8.0 (12.4)	8.5 (13.1)	1.000
	Max hip flexion/extension	52.7 (12.6)	53.1 (9.5)	1.000
	Min hip adduction/abduction	3.9 (9.5)	3.2 (9.1)	1.000
	Max hip adduction/abduction	20.9 (8.8)	23.4 (9.0)	1.000
Range of motion [deg], mean (SD)	Ankle dorsi-/plantarflexion	43.4 (12.0)	44.0 (10.8)	1.000
	Knee flexion/extension	96.9 (27.1)	106.6 (22.0)	0.073
	Hip flexion/extension	44.5 (14.6)	44.8 (15.1)	1.000
	Hip adduction/abduction	26.5 (6.5)	24.9 (6.9)	1.000
Displacement [m], mean (SD)	Horizontal	0.07 (0.03)	0.06 (0.02)	0.286
	Vertical	0.03 (0.02)	0.03 (0.02)	1.000
	Lateral	0.05 (0.01)	0.05 (0.01)	1.000
ACC, mean (SD)	Ankle/knee	0.91 (0.09)	0.92 (0.09)	1.000
	Knee/hip	0.95 (0.09)	0.95 (0.08)	1.000

Abbreviations: s: seconds; SD: standard deviation; deg: degrees; m: meters; ACC: angular component of coefficient of correspondence.

**Table A3:** Characteristics of clusters with the corresponding *p*-values calculated with the Mann–Whitney U test for continuous and the Fisher’s Exact Test for categorical variables.

Cohort	Cluster 1	Cluster 2	*p*-Value
Number, *n*	15	15	
Age [years], mean (SD)	59.3 (11.6)	51.5 (15.1)	0.164
Sex [female], *n* (%)	3 (20.0)	6 (40.0)	0.427
BMI [kg/m^2^]	25.4 (3.5)	24.8 (4.4)	0.534
AIS D, *n* (%)	15 (100.0)	15 (100.0)	<1.000
NLI, *n* (%)	5 (33.3) cervical 10 (66.7) thoracic	7 (46.7) cervical 8 (53.3) thoracic	0.710
Etiology, *n* (%)	5 (33.3) traumatic 10 (66.7) non-traumatic	6 (40.0) traumatic 9 (60.0) non-traumatic	<1.000
SCIM Mob., mean (SD)	37.5 (2.5)	32.9 (6.1)	0.026

Abbreviations: BMI: body mass index; AIS: ASIA Impairment Scale; NLI: neurological level of injury; SCIM Mob.: mobility subscale of the Spinal Cord Independence Measure III.
